# Draft Genome Sequences of 11 Rhodopsin Gene-Containing Actinobacteria (*Geodermatophilaceae*) from Saline Arid Habitats in the Central Asian Deserts

**DOI:** 10.1128/mra.01368-22

**Published:** 2023-04-17

**Authors:** Sergey V. Tarlachkov, Irina P. Starodumova, Olga V. Boueva, Lyudmila V. Lysak, Sergei A. Subbotin, Lyudmila I. Evtushenko

**Affiliations:** a All-Russian Collection of Microorganisms (VKM), G. K. Skryabin Institute of Biochemistry and Physiology of Microorganisms, Pushchino Scientific Center for Biological Research, Russian Academy of Sciences, Pushchino, Russia; b Branch of Shemyakin-Ovchinnikov Institute of Bioorganic Chemistry, Russian Academy of Sciences, Pushchino, Russia; c Shemyakin-Ovchinnikov Institute of Bioorganic Chemistry, Russian Academy of Sciences, Moscow, Russia; d Soil Science Faculty, Lomonosov Moscow State University, Moscow, Russia; e California Department of Food and Agriculture, Sacramento, California, USA; f Center of Parasitology, A. N. Severtsov Institute of Ecology and Evolution, Russian Academy of Sciences, Moscow, Russia; Wellesley College

## Abstract

Draft genome sequences of 11 strains of putative new species of *Geodermatophilaceae* were generated using Illumina technology. The genome sizes ranged from 4.19 to 4.99 Mb, with G+C contents of 73.5% to 74.6%, and contained genes for microbial rhodopsins. This study will contribute to our knowledge of the ecology and diversity of members of the family *Geodermatophilaceae*.

## ANNOUNCEMENT

The family *Geodermatophilaceae* (phylum *Actinobacteria*) includes four genera with validly published names, *viz*., *Blastococcus*, *Geodermatophilus*, *Klenkia*, and *Modestobacter* (https://lpsn.dsmz.de/family/geodermatophilaceae). Members of this family are known as aerobic heterotrophs capable of growing in media with low levels of nutrients, but microaerophilic representatives have also been reported among them (1–3). They occur in different ecosystems, often inhabiting extreme ones, can be pioneers in the colonization of exiguous substrates, and play a critical role in determining the bacterial community structure in deserts (1–3). Recently, microbial rhodopsins have been described in members of this family (4).

Strains used in this work were isolated between 1991 and 1993 from desert plants and salt crust on the soil surface (Table 1) and maintained for nearly 30 years in the All-Russian Collection of Microorganisms (VKM) before the study. The isolation was conducted by serial dilution plating on medium containing glucose (1.0 g), peptone (2.0 g), yeast extract (1.0 g), casein hydrolysate (1.0 g), malt extract (1.0 g), glycerol (10.0 mL), CaCO_3_ (5.0 g), agar (20.0 g), and tap water (1 L), pH 7.0 to 7.2.

**TABLE 1:** Strain characteristics, sequencing data, and DDBJ/ENA/GenBank accession numbers of the genome sequences in this study

Organism	Source^*a*^	Geographical location	No. of reads	Coverage (×)	No. of scaffolds	Scaffold *N*_50_ (bp)	Genome size (Mbp)	G+C content (%)	No. of proteins	GenBank accession no.	SRA accession no.
*Modestobacter* sp. VKM Ac-2977	Salt crust	Kyzylkum Desert, Uzbekistan	16,153,904	547	16	1,097,579	4.31	73.8	4,058	JAPVBQ000000000	SRR22840112
*Modestobacter* sp. VKM Ac-2978	Salt crust	Kyzylkum Desert, Uzbekistan	15,928,698	471	25	475,612	4.95	73.6	4,665	JAPVBP000000000	SRR22840111
*Modestobacter* sp. VKM Ac-2979	Saltwort	Kyzylkum Desert, Uzbekistan	14,499,326	421	14	576,049	4.99	73.5	4,679	JAPVBO000000000	SRR22840109
*Modestobacter* sp. VKM Ac-2980	Saltwort	Kyzylkum Desert, Uzbekistan	14,674,592	427	12	767,119	4.99	73.5	4,684	JAPVBN000000000	SRR22840108
*Modestobacter* sp. VKM Ac-2981	Salt crust	Kyzylkum Desert, Uzbekistan	14,081,214	431	17	987,659	4.78	73.8	4,439	JAPVBM000000000	SRR22840107
*Modestobacter* sp. VKM Ac-2982	Salt crust	Kyzylkum Desert, Uzbekistan	18,327,836	561	14	803,233	4.78	73.8	4,436	JAPVBL000000000	SRR22840106
*Modestobacter* sp. VKM Ac-2983	Saltwort	Kyzylkum Desert, Uzbekistan	15,649,426	503	12	1,131,808	4.51	73.8	4,233	JAPVBK000000000	SRR22840105
*Modestobacter* sp. VKM Ac-2984	Salt crust	Kyzylkum Desert, Uzbekistan	15,666,186	501	21	383,856	4.56	73.8	4,228	JAPVBJ000000000	SRR22840104
*Modestobacter* sp. VKM Ac-2985	Salt crust	Karakum Desert, Turkmenistan	18,313,752	563	9	1,419,625	4.72	73.9	4,381	JAPVBI000000000	SRR22840103
*Modestobacter* sp. VKM Ac-2986	Salt crust	Kyzylkum Desert, Uzbekistan	16,034,602	557	15	679,792	4.19	74.6	3,911	JAPVBH000000000	SRR22840102
*Blastococcus* sp. VKM Ac-2987	Salt crust	Kyzylkum Desert, Uzbekistan	14,560,286	460	12	1,332,385	4.61	74.2	4,358	JAPVBG000000000	SRR22840110

a The salt crust was found on the soil surface.

For genome sequencing, biomass was grown in liquid PYG medium (peptone, 5.0 g; yeast extract, 3.0 g; glucose, 5.0 g; KH_2_PO_4_, 0.2 g; distilled water, 1.0 L; рН 7.2) at 28°C with shaking at 180 rpm for 2 to 3 days. Genomic DNA was extracted from the pure culture using a QIAamp DNA minikit (Qiagen, Germany), following the manufacturer’s protocol for isolation of genomic DNA from Gram-positive bacteria. Prior to adding ethanol to the sample, undissolved cell debris was removed by centrifugation. DNA library construction and sequencing were performed by Novogene Co., Ltd. Libraries were generated using the NEBNext DNA library prep kit for Illumina (New England Biolabs, USA), following the manufacturer’s recommendations. The pooled DNA libraries were sequenced on an Illumina NovaSeq 6000 instrument to obtain 150-bp paired-end reads.

The quality of the reads was checked using FastQC v0.11.8 (https://www.bioinformatics.babraham.ac.uk/projects/fastqc/). Adapter sequences and low-quality regions in the raw reads were trimmed using Trimmomatic v0.39 (5). The trimmed reads were assembled using SPAdes v3.15.4 (6). The assemblies were annotated using NCBI PGAP v6.3 (7). The genome relatedness indices, such as the average nucleotide identity (ANI) and digital DNA-DNA hybridization (dDDH) values, were calculated using the JSpecies v1.2.1 (8) and GGDC v3.0 (9) tools, respectively. The accession numbers and characteristics of the genomes are provided in Table 1.

The determined ANI and dDDH values showed that strain VKM Ac-2987 was most closely related to Blastococcus xanthinilyticus DSM 46842^T^ (ANI, 84.5%; dDDH, 28.8%) and VKM Ac-2986 was closest to Modestobacter muralis DSM 100205^T^ (ANI, 92.1%; dDDH, 47.5%), while the other nine strains exhibited the closest relatedness to Modestobacter caceresii KNN 45-2b^T^ (ANI, 90.5% to 95.1%; dDDH, 42.6% to 62.4%), originating from an extreme hyperarid Atacama Desert soil (10). The above ANI and dDDH values were below the borderlines for species delineation (11), indicating that all our strains belong to putative new species. It is also worth noting that these strains were found to contain genes for microbial rhodopsins.

Further biochemical and physiological study of these strains, accompanied by functional genomics and proteomics data, will expand our knowledge of the survival mechanisms of members of the family *Geodermatophilaceae* under multiple environmental pressures in arid and saline ecosystems.

### Data availability.

These whole-genome shotgun sequencing projects have been deposited at DDBJ/ENA/GenBank under the accession numbers listed in Table 1.
